# Tetrabutylammonium Bromide (TBAB) Catalyzed Synthesis of Bioactive Heterocycles

**DOI:** 10.3390/molecules25245918

**Published:** 2020-12-14

**Authors:** Bimal Krishna Banik, Bubun Banerjee, Gurpreet Kaur, Shivam Saroch, Rajat Kumar

**Affiliations:** 1 Department of Mathematics and Natural Sciences, College of Sciences and Human Studies, Deanship of Research Development, Prince Mohammad Bin Fahd University, Al Khobar 31952, Saudi Arabia; 2 Department of Chemistry, Indus International University, V.P.O. Bathu, Distt. Una, Himachal Pradesh 174301, India or bubun.banerjee@iiuedu.in (B.B.); kaur80328@gmail.com (G.K.); shivamsaroch14@gmail.com (S.S.); ra131997@gmail.com (R.K.)

**Keywords:** tetrabutylammonium bromide, TBAB, phase-transfer catalyst, metal-free synthesis, bioactive heterocycles

## Abstract

During the last two decades, tetrabutylammonium bromide (TBAB) has gained significant attention as an efficient metal-free homogeneous phase-transfer catalyst. A catalytic amount of TBAB is sufficient to catalyze various alkylation, oxidation, reduction, and esterification processes. It is also employed as an efficient co-catalyst for numerous coupling reactions. It has also acted as an efficient zwitterionic solvent in many organic transformations under molten conditions. In this review, we have summarized the recent developments on TBAB-catalyzed protocols for the efficient synthesis of various biologically promising heterocyclic scaffolds.

## 1. Introduction

**Figure 1:**
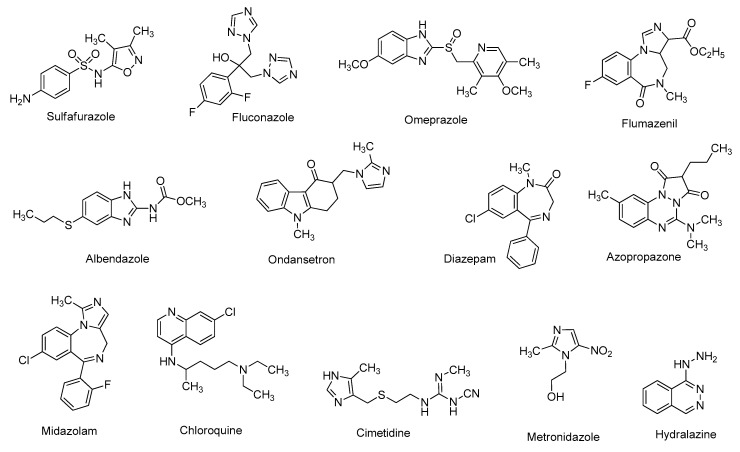
Glimpse of marketed drugs containing heterocycles.

Heterocyclic skeletons are very common in commercially available drug molecules (Figure 1) [1]. Heterocycles are the main building blocks of many naturally occurring compounds [2]. Various synthetic heterocyclic scaffolds have been found to possess a wide range of biological efficacies, including anti-inflammatory [3], anti-malarial [4], anti-tubercular [5], anti-cancer [6], anti-asthmatic [7], anti-histaminic [8], anti-hypertensive [9], anti-depressant [10], anti-microbial [11], anti-rheumatic [12], anti-diabetic [13], anti-Alzheimer’s, anti-Parkinson’s, anti-Huntington’s disease [14], and many more activities [15,16].

For the synthesis of diverse heterocyclic entities, the screening of suitable catalysts plays an important role [17]. At present, scientists prefer metal-free organocatalysts in order to avoid metal contamination in the synthesized products. As a result, during the last decade, various organocatalysts have gained a great deal of attention in carrying out organic transformations under environmentally benign conditions [18,19,20]. Among many others, metal-free phase-transfer catalysts are being widely used in various organic reactions due to their ecofriendly, mild, and biocompatible nature [21]. Various phase-transfer catalysts showed immense activity in reactions where a reactant soluble in the organic phase needs to react with an anionic reactant soluble in the aqueous phase.

Recently, tetrabutylammonium bromide (TBAB) has gained tremendous attention as an efficient homogeneous phase-transfer catalyst. TBAB is an environmentally benign, non-volatile, non-flammable, non-corrosive, low-cost, commercially available ammonium salt with high thermal and chemical stability [22]. In TBAB, tetrabutylammonium salt can dissolve in both aqueous as well as in organic solvents, which helps to transport the water-soluble anionic reactants into the organic phase. Moreover, molten TBAB was also employed as an efficient ionic liquid to carry out organic transformations under solvent-free conditions [23,24,25]. In some reactions, it was observed that the addition of a catalytic amount of TBAB as co-catalyst enhanced the reaction rate as well as product yields [26,27,28,29,30]. The abovementioned unique capabilities of TBAB make this catalyst very attractive. In many occasions, normal monophasic catalysts either failed to carry out such reactions or afforded poor yields. As a result, the catalytic activity of TBAB has been continuously explored for various reactions. It showed excellent catalytic efficacies for the synthesis of *N*-aryl amines [31] and 1-alkyl/aryl-2-(1-arylsulfonyl alkyl) benzimidazoles [32]. It was also employed for the carbonylation-peroxidation of styrene derivatives [33], the alkylation of aldehydes or ketones [34], the *S*-alkylation of 4-mercapto-6-methyl-2-pyrone [35], the *N-*alkylation of acridones [36], the sulfonylation of *para*-quinone methides [37], Suzuki cross-coupling reaction [38], Heck reaction [39], and Suzuki–Miyaura reaction [40].

In the following sections, we will discuss various TBAB-catalyzed synthetic approaches which have been reported for the preparation of diverse biologically relevant heterocycles reported.

## 2. Applications of TBAB for the Synthesis of Bioactive *N*-Heterocycles

### 2.1. Synthesis of 1,4-Dihydropyridines

**Scheme 1:**
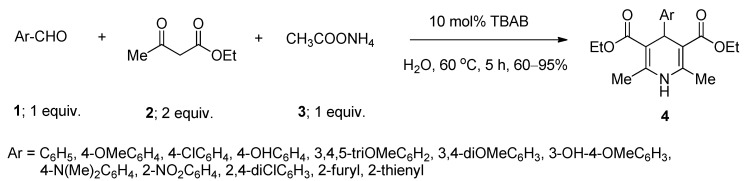
TBAB-catalyzed synthesis of 1,4-dihydropyridines in water at 60 °C.

1,4-Dihydropyridine and related derivatives are found to possess a wide range of biological efficacies, including anti-bacterial [41], anti-diabetic [42], anti-cancer [43], anti-HIV [44], anti-convulsant [45], and anti-tubercular [46] activities. A number of methods were reported for the synthesis of these biologically significant scaffolds using various homogeneous as well as heterogeneous catalysts [47,48,49,50], ionic liquids [51], and fluorinated solvents [52]. The use of metal-containing catalysts and toxic solvents are some of the major drawbacks of these reported protocols. In 2014, Kumar et al. [53] developed a facile method for the synthesis of a series of 1,4-dihydropyridine derivatives via one-pot pseudo four-component Hantzsch reaction between one equivalent of various aryl or heteroaryl aldehydes (**1**), two equivalents of ethyl acetoacetate (**2**), and one equivalent of ammonium acetate (**3**) using 10 mol% of TBAB as an efficient phase-transfer catalyst in aqueous medium at 60 °C (Scheme 1). Structurally diverse aromatic aldehydes produced the desired products with excellent yields. Heteroaryl aldehydes also smoothly underwent the reaction and yielded the desired products. Under the same optimized condition, the reactions afforded comparable yields by using benzyltriethyl ammonium chloride as catalyst whereas lower yields were obtained with cetyltrimethylammonium bromide as catalyst.

### 2.2. Synthesis of 2-Substituted Imidazolines

**Scheme 2:**
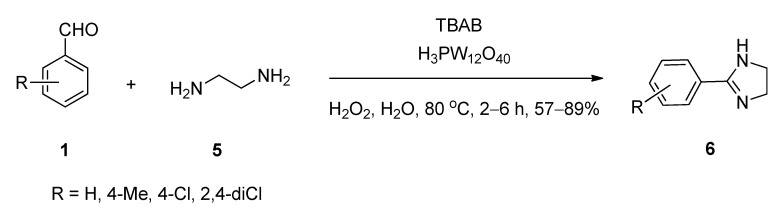
TBAB co-catalyzed synthesis of 2-substituted imidazolines in water.

**Scheme 3:**
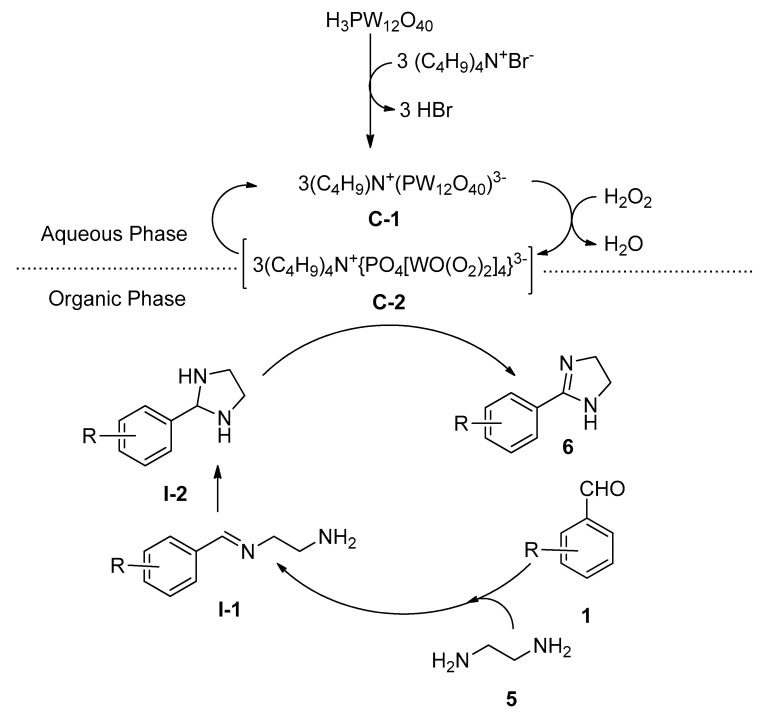
Plausible role of tungstophosphoric acid and TBAB as an efficient combined phase-transfer catalytic system for the synthesis of 2-substituted imidazolines in water.

Liu et al. [54] reported a facile and eco-friendly method for the efficient synthesis of 2-substituted imidazolines (**6**) starting from aromatic aldehydes (**1**) and ethylenediamine (**5**) in the presence of a catalytic amount of both tungstophosphoric acid as well as tetrabutylammonium bromide as an efficient phase-transfer catalytic system using hydrogen peroxide as oxidant in water at 80 °C (Scheme 2). During optimization, a lower yield was recorded in the absence of TBABI (i.e., using only tungstophosphoric acid as catalyst). A probable role of the dual catalysts is shown in Scheme 3. In the organic phase, the rapid condensation of benzaldehyde and ethylenediamine produced intermediate **I-1**, which after cyclization produced the second intermediate **I-2**. In the aqueous phase, the combination of a catalytic amount of TBAB and tungstophosphoric acid generated a novel complex **C-1** which in the presence of H_2_O_2_ yielded peroxo complex **C-2**. This in-situ generated complex can catalyze the formation of the desired 2-phenylimidazoline **5** from the intermediate **I-2** by entering the organic phase.

### 2.3. Synthesis of 2,4,5-Triaryl Imidazoles

**Scheme 4:**
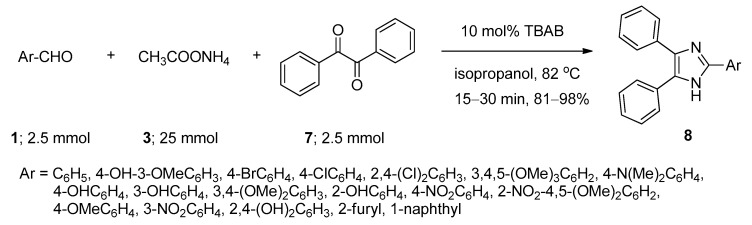
TBAB-catalyzed synthesis of 2,4,5-triaryl imidazoles in isopropanol at 82 °C.

**Scheme 5:**
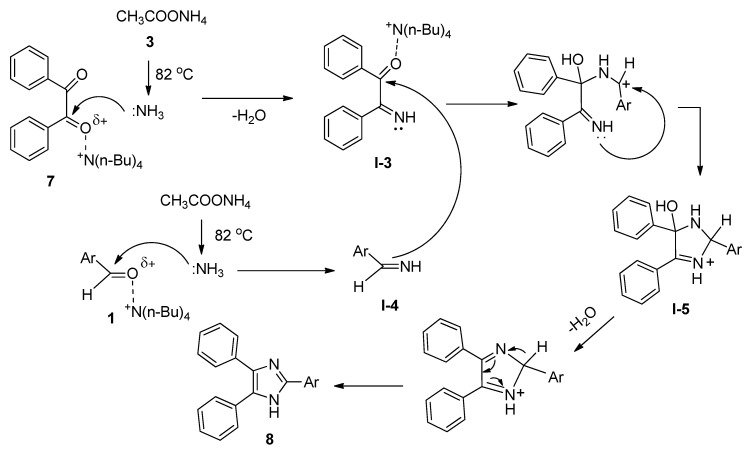
Plausible mechanism of TBAB-promoted synthesis of 2,4,5-triaryl imidazoles.

2,4,5-Trisubstituted imidazoles have been found to possess immense biological activities [55,56,57,58,59]. 2,4,5-Triphenyl imidazole (**8**) was first synthesized in 1882 from the reaction of aryl aldehyde (**1**) and benzyl (**7**) in alcoholic ammonia solution [60]. Later on, a number of methods were reported involving ammonium acetate (**3**) as the source of ammonia in the presence of various Lewis acidic metal salts as catalyst [61,62,63,64,65,66,67]. These reported methods suffered from many drawbacks, such as harsh conditions, use of metal-containing catalysts, and lower yields. Starting from the same batch of reactants, in 2008, Chary et al. [68] developed a facile, efficient, and environmentally benign protocol for the synthesis of 2,4,5-triaryl imidazoles (**8**) using a catalytic amount of TBAB isopropanol under reflux conditions (Scheme 4). After completion of the reaction, TBAB-containing reaction medium was reused for a further run. Aldehydes with both electron-donating as well as electron-withdrawing substituents afforded the desired products with excellent yields. All the reactions were completed within thirty minutes. A probable mechanistic approach is outlined in Scheme 5. TBAB activated the carbonyl group of benzil, which facilitated the formation of intermediate **I-3** by the attack of ammonium generated from ammonium acetate. On the other hand, corresponding Schiff bases (**I-4**) were also prepared from the reaction between aromatic aldehydes and in-situ-generated ammonia in the presence of TBAB as catalyst. Now, the combination of **I-3** and **I-4** produced another intermediate **I-5** which on further dehydration followed by aromatization yielded the desired 2,4,5-triaryl imidazoles (**8**).

### 2.4. Synthesis of 1,3-Dihydrobenzimidazol-2-Ones

**Scheme 6:**
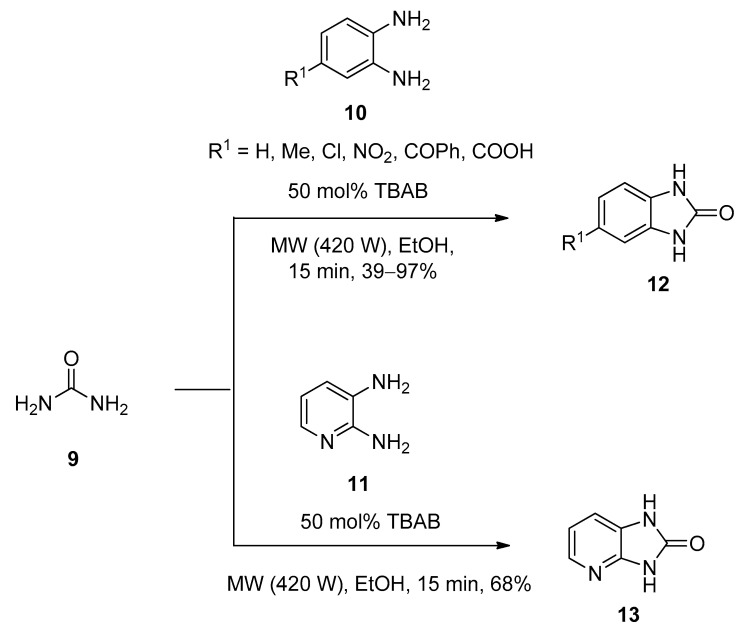
TBAB-catalyzed synthesis of 1,3-dihydrobenzimidazol-2-ones and 1*H*-imidazo[4,5-*b*]pyridin-2(3*H*)-one under microwave-assisted conditions.

Aghapoor et al. [69] developed a simple, rapid, microwave-assisted protocol for the efficient synthesis of 1,3-dihydrobenzimidazol-2-ones (**12**) from the reactions of urea (**9**) and *o*-phenylenediamines (**10**) using TBAB as a catalyst in ethanol. Under the optimized reaction conditions, pyridine-2,3-diamine (**11**) also reacted with urea (**9**) and afforded the corresponding 1*H*-imidazo [4,5-*b*]pyridin-2(3*H*)-one (**13**) with 68% yield (Scheme 6). All the reactions were completed within just fifteen minutes. *o*-Phenylenediamines with both electron-donating as well as electron-withdrawing substituents produced the desired products with good yields. Although the exact mechanism was not discussed in the mother literature, we can assume that TBAB initiates the reaction by activating the carbonyl carbon of urea.

### 2.5. Synthesis of Pyrrolo[2,3-d]pyrimidine Derivatives

**Scheme 7:**
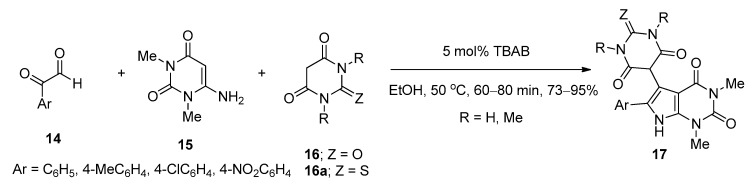
TBAB-catalyzed synthesis of pyrrolo[2,3-*d*]pyrimidine derivatives in ethanol.

**Scheme 8:**
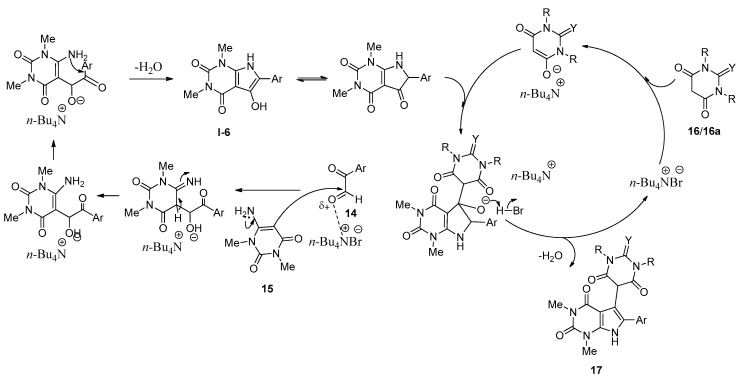
Plausible mechanism of TBAB-catalyzed synthesis of pyrrolo[2,3-*d*]pyrimidine derivatives.

A straightforward one-pot three-component reaction protocol was reported utilizing TBAB as the catalyst. Using this protocol, a series of densely functionalized pyrrolo[2,3-*d*]pyrimidine derivatives (**17**) were successfully synthesized from the reactions of aryl glyoxals (**14**), 6-amino-1,3-dimethyluracil (**15**), and barbituric acid (**16**) or thiobarbituric acid derivatives (**16a**) in ethanol at 50 °C (Scheme 7) [70]. Clean reaction profile, excellent yields, short reaction times, and easy work-up procedure are some of the major advantages of this protocol. The plausible mechanism of this reaction is shown in Scheme 8. TBAB initiated the reaction by activating the aldehydic carbon of aryl glyoxals. Reaction of aryl glyoxals and 6-amino-1,3-dimethyluracil (**15**) yielded intermediates **I-6**, which then further reacted with barbituric acid (**16**) or thiobarbituric acid derivatives (**16a**) to produce the desired products **17**.

### 2.6. Synthesis of 2,3-Dihydroquinazolin-4(1H)-Ones

**Scheme 9:**
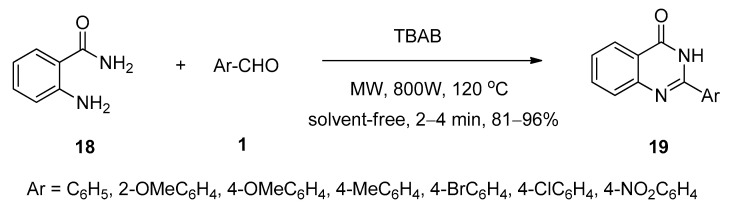
TBAB-catalyzed synthesis of 2,3-dihydroquinazolin-4(1*H*)-ones under microwave-irradiated solvent-free conditions.

Heterocycles with quinazolin-4-one skeleton have been found to possess a broad range of biological activities [71,72,73,74]. Davoodnia et al. [75] achieved the synthesis of a series of 2-arylquinazolin-4(3*H*)-ones (**19**) via the cyclocondensation reactions between 2-aminobenzamide (**18**) and aromatic aldehydes (**1**) in the presence of a catalytic amount of tetrabutylammonium bromide under microwave-assisted solvent-free conditions at 120 °C (Scheme 9). All the reactions were completed within just four minutes. The desired products were isolated with excellent yields.

### 2.7. Synthesis of 1,5-Benzodiazepine Derivatives

**Scheme 10:**
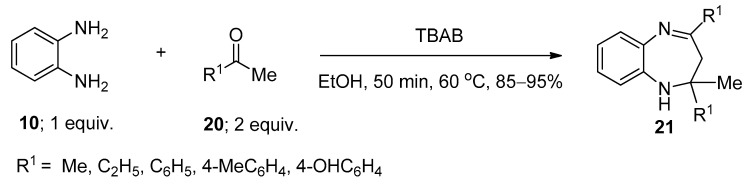
TBAB-catalyzed synthesis of 1,5-benzodiazepine derivatives in ethanol.

**Scheme 11:**
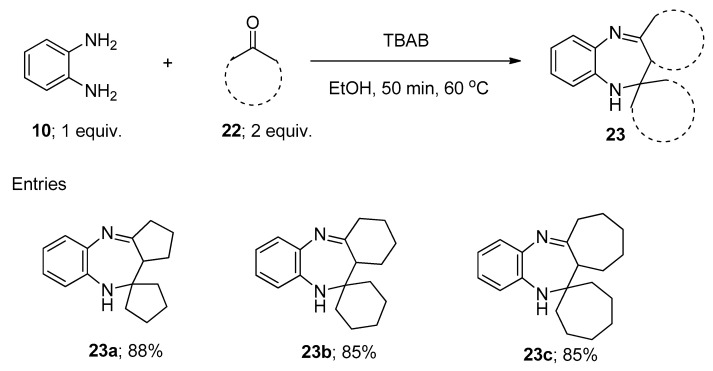
TBAB-catalyzed synthesis of spiro-benzodiazepine derivative.

Benzodiazepine and related derivatives showed potent pharmaceutical activities [76]. Among many others, 2,3-dihydro-1*H*-1,5-benzodiazepines (**21**) gained much attention. A number of methods were reported for the synthesis of these biologically promising scaffolds utilizing numerous homogeneous as well as heterogeneous catalysts, such as BF_3_OEt [77], Ag_3_PW_12_O_40_ [78], PPA-SiO_2_ [79], zinc montmorillonite [80], Yb(OTf)_3_ [81], MgO-POCl_3_ [82], Amberlyst [83], and superacid sulphated zirconia [84]. The use of a toxic and costly catalyst is the common drawback of these reported methods. In 2012, Baseer and Khan [85] synthesized a series of structurally diverse 2,3-dihydro-1*H*-1,5-benzodiazepines (**21**) from the reactions of one equivalent of *o*-phenylenediamine (**10**) and two equivalents of various acyclic ketones (**20**) using TBAB as an efficient catalyst in ethanol at 60 °C (Scheme 10). Under the same optimized conditions, they were also able to synthesize a series of spiro-benzodiazepine derivatives (**23**) using cyclic ketones (**22**) instead of acyclic ketones (Scheme 11). In all cases, excellent yields of the desired products were obtained through the formation of Schiff bases followed by a cyclization pathway.

### 2.8. Synthesis of 5-Substituted 1H-Tetrazoles

**Scheme 12:**
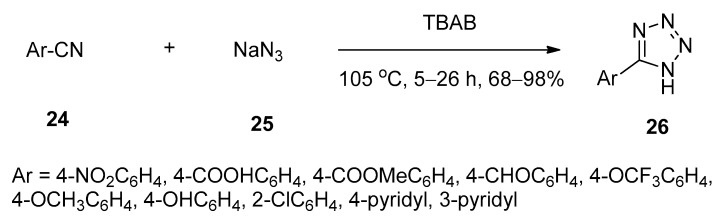
TBAB-promoted synthesis of 5-substituted 1*H* -tetrazoles.

Xie et al. [86] reported a simple and efficient protocol for the synthesis of a series of 5-substituted 1*H*-tetrazoles (**26**) with excellent yields from the reactions of aryl nitrile (**24**) and sodium azide (**25**) in molten tetrabutylammonium bromide at 105 °C (Scheme 12). In this reaction, molten TBAB played a dual role, both as a solvent as well as a catalyst. Under this condition, aliphatic nitrile failed to produce the desired products. Here, TBAB polarized the cyano group, which facilitated the attack by the azide ion.

## 3. Applications of TBAB for the Synthesis of Bioactive *O*-Heterocycles

### 3.1. Synthesis of 3-Nitro-2H-Chromenes

**Scheme 13:**
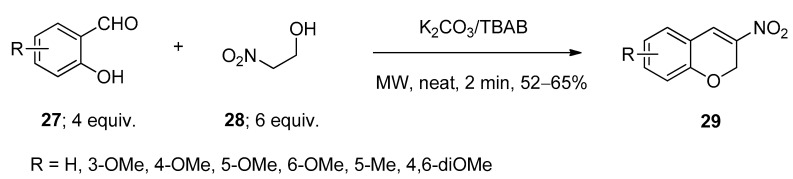
TBAB-catalyzed synthesis of 3-nitro-2*H*-chromenes under microwave-assisted conditions.

Synthesis of 3-nitro-2*H*-chromenes (**29**) was achieved via the microwave-assisted reactions of substituted salicylaldehydes (**27**) and 2-nitro ethanol (**28**) using anhydrous potassium carbonate as base in the presence of a catalytic amount of TBAB as catalyst under solvent-free conditions (Scheme 13) [87]. The presence of a base facilitated the formation of carbanion on the carbon atom attached with a nitro group. After being activated by TBAB, the aldehydic group underwent condensation with the in-situ-generated carbanion and the resulting intermediate after cyclization yielded the corresponding desired products **29**.

### 3.2. Synthesis of 3,4-Dihydropyrano[c]chromene

**Scheme 14:**
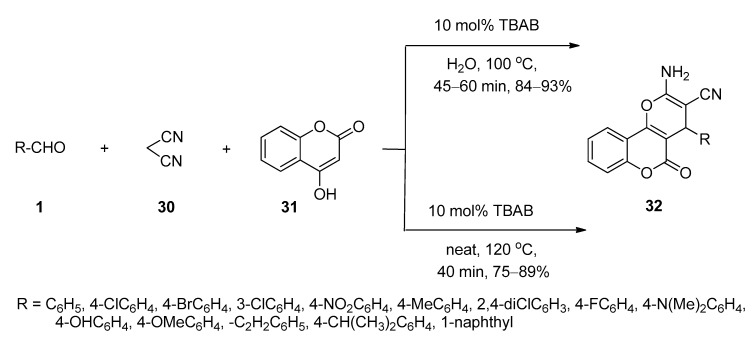
TBAB-catalyzed synthesis of 3,4-dihydropyrano[*c*]chromenes in water or solvent-free conditions.

**Scheme 15:**
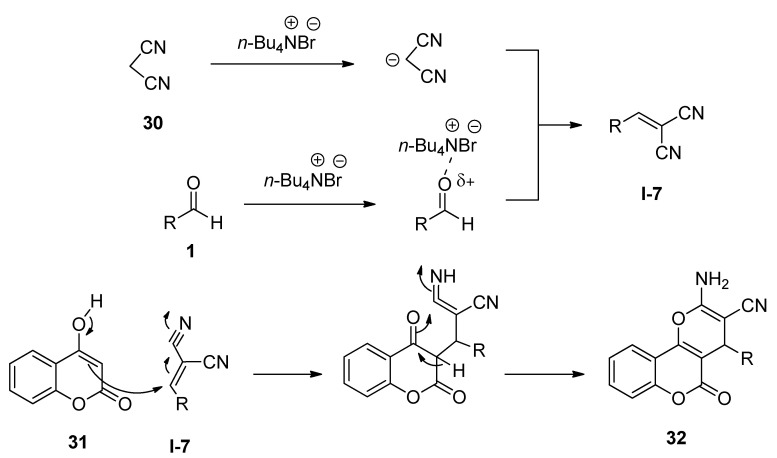
Plausible mechanism of TBAB-catalyzed synthesis of 3,4-dihydropyrano[*c*]chromenes.

3,4-Dihydropyrano[*c*]chromenes and related derivatives have been found to possess a wide range of biological activities [88,89,90]. In 2009, Khurana and Kumar [91] reported a facile protocol for the synthesis of 3,4-dihydropyrano[*c*]chromenes (**32**) via one-pot three-component reactions of various aldehydes (**1**), malononitrile (**30**), and 4-hydroxycoumarin (**31**) in the presence of a catalytic amount of TBAB as an efficient catalyst in water at 100 °C (Scheme 14). The same batches of reactions also afforded excellent yields of the desired products under solvent-free conditions at 120 °C. Aldehydes (**1**) in the presence of TBAB underwent Knoevenagel condensation with malononitrile (**30**) and generated the corresponding intermediate **I-7** which then further reacted with 4-hydroxycoumarin (**31**) to produce the desired product **32** (Scheme 15).

### 3.3. Synthesis of 4-Phenyl-1,3-Dioxolan-2-One

**Scheme 16:**
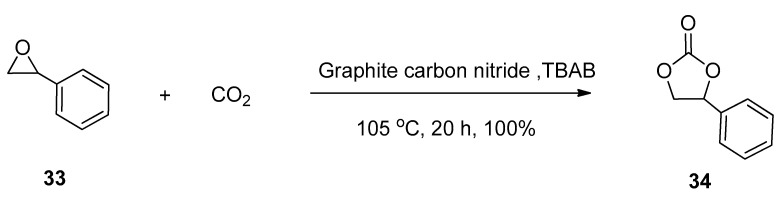
Combination of graphite carbon nitride and TBAB-catalyzed synthesis of 4-phenyl-1,3-dioxolan-2-one.

A combination of graphite carbon nitride and TBAB was used as an efficient catalytic system for the synthesis of 4-phenyl-1,3-dioxolan-2-one (**34**) from 2-phenyloxirane (**33**) under carbon-dioxide-filled reaction conditions at 105 °C (Scheme 16) [92]. Although the reaction took twenty hours to complete, it produced 100% yield of the desired product.

### 3.4. Synthesis of Isocoumarin-1-Imines and Isobenzofuran-1-Imines

**Scheme 17:**
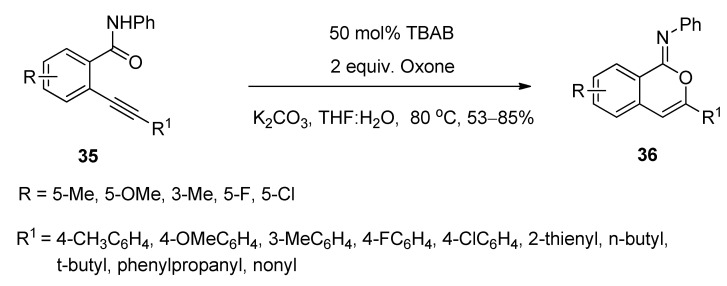
TBAB-catalyzed synthesis of isocoumarin-1-imines via 6-*endo-dig oxy*-cyclization 2-alkynylbenzamide.

**Scheme 18:**
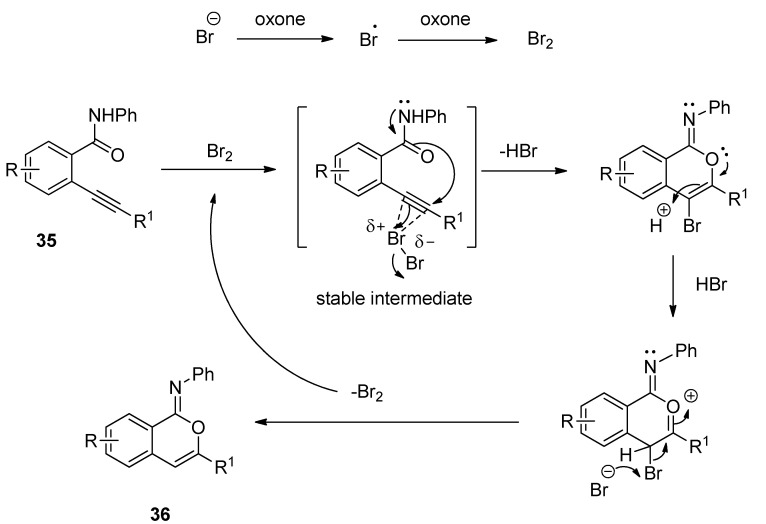
Plausible mechanism of the formation of isocoumarin-1-imines via 6-*endo-dig oxy*-cyclization 2-alkynylbenzamide.

**Scheme 19:**
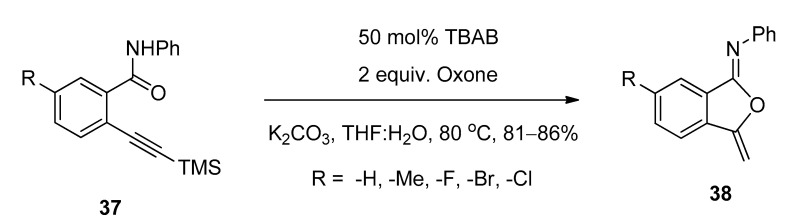
TBAB-catalyzed synthesis of isobenzofuran-1-imines starting from *N*-phenyl 2-trimethylsilylethynylbenzamides.

**Scheme 20:**
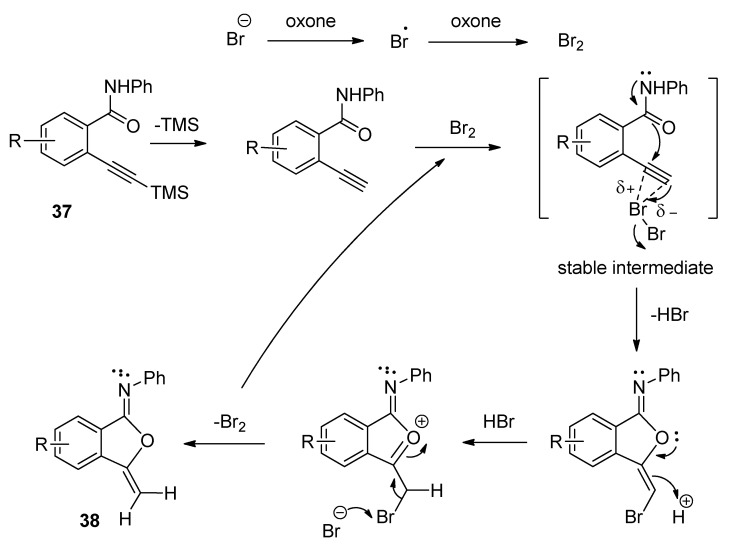
Plausible mechanism of the formation of isobenzofuran-1-imines starting from *N*-phenyl 2-trimethylsilylethynylbenzamides.

A facile and regioselective TBAB-catalyzed efficient protocol was reported for the synthesis of a series of isocoumarin-1-imines (**36**) through the 6-*endo-dig oxy*-cyclization 2-alkynylbenzamide (**35**) (Scheme 17) [93]. The reactions were carried out using two equivalents of Oxone as oxidizing agent in the presence of potassium carbonate as base in THF-water mixture as solvent at 80 °C. The plausible mechanism of the transformation is shown in Scheme 18. Under the same optimized conditions, when *N*-phenyl 2-trimethylsilylethynylbenzamides (**37**) were used as starting components, the corresponding isobenzofuran-1-imines (**38**) were isolated with excellent yields (Scheme 19). The probable mechanistic approach of this transformation is shown in Scheme 20.

### 3.5. Synthesis of Tetrahydrobenzo[b]pyran

**Scheme 21:**
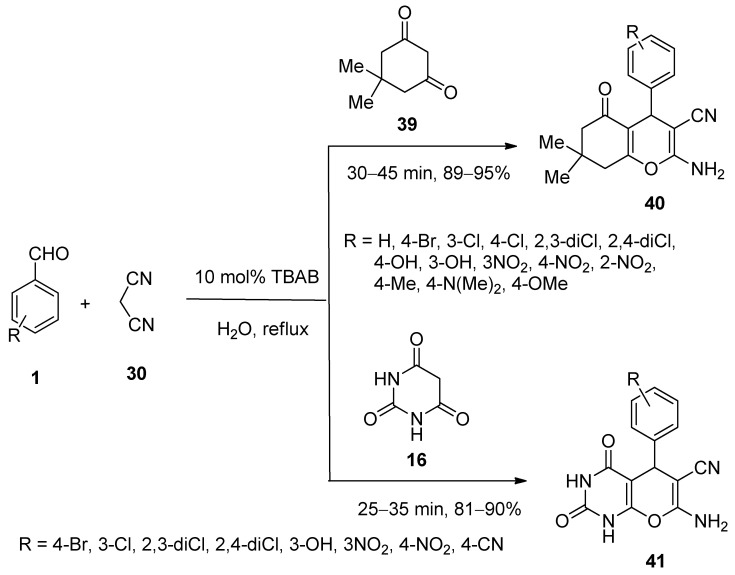
TBAB-catalyzed synthesis of tetrahydrobenzo[*b*]pyran and pyrano[2,3-*d*]pyrimidinone derivatives in water.

Many tetrahydrobenzo[*b*]pyrans are being used as anti-cancer, anti-coagulant, anti-anaphylactic, diuretic, and spasmolytic agents [94,95,96]. In 2010, Mobinikhaledi and Fard [97] developed a mild, efficient, and convenient protocol for the synthesis of a number of tetrahydrobenzo[*b*]pyran derivatives (**40**) via one-pot three-component reactions of substituted benzaldehydes (**1**), malononitrile (**30**), and dimedone (**39**) in the presence of a catalytic amount of TBAB as catalyst in aqueous medium under reflux conditions (Scheme 21). Substituted benzaldehydes also proceeded smoothly and afforded the desired products with excellent yields. Under the same optimized conditions, they were also synthesized as a series of pyrano[2,3-*d*]pyrimidinone derivatives (**41**) using barbituric acid (**16**) instead of dimedone (**39**) (Scheme 21). Excellent yields, mild reaction conditions, water as solvent, and easy purification procedure are some of the major advantages of this protocol.

### 3.6. Synthesis of Xanthones

**Scheme 22:**
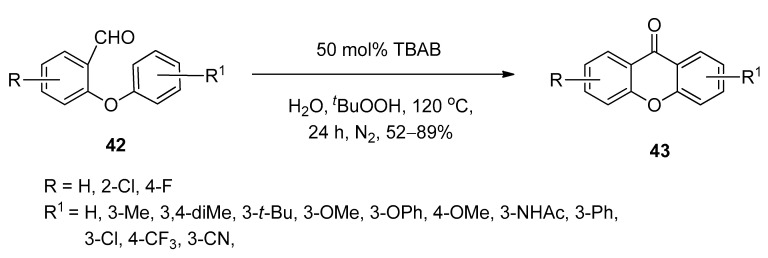
TBAB-catalyzed synthesis of xanthones from 2-aryloxybenzaldehydes in water at 120 °C.

**Scheme 23:**
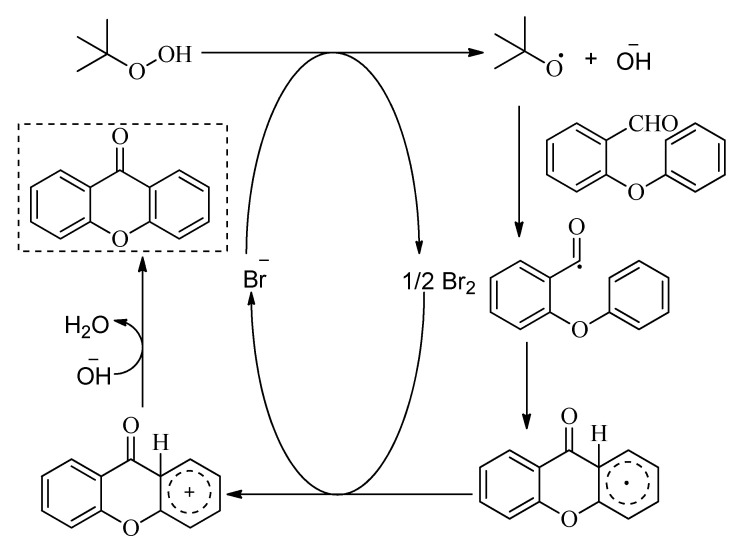
Plausible mechanism of the formation of xanthones from 2-aryloxybenzaldehydes using TBAB as catalyst.

Rao et al. [98] developed a simple and straightforward aqueous-mediated protocol for the intramolecular annulations of 2-aryloxybenzaldehydes (**42**) which afforded the corresponding xanthones (**43**) with moderate to excellent yields using *tert*-butyl hydroperoxide (TBHP) as an oxidant in the presence of TBAB as catalyst at 120 °C (Scheme 22). The plausible mechanism of this transformation is shown in Scheme 23. The reaction proceeded through the direct oxidative coupling reactions of C-H bonds of aldehydes and aromatic C-H bonds. Under the same optimized reaction conditions, 2-aryloxybenzaldehydes with both electron-donating as well as electron-withdrawing substituents showed good tolerance and yielded the corresponding desired products.

### 3.7. Synthesis of Aryl-14H-Dibenzo[a.j]xanthenes

**Scheme 24:**
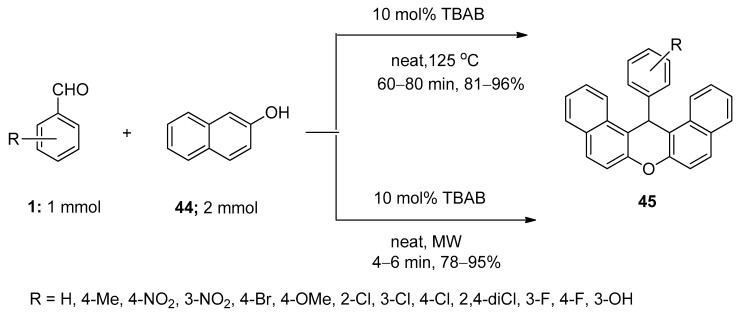
TBAB-catalyzed synthesis of aryl-14*H*-dibenzo[*a*,*j*]xanthenes under solvent-free conditions.

Benzoxanthene and its congeners have shown various biological activities [99,100,101,102]. In 2008, Kantevari et al. [103] reported a facile method for the synthesis of a series of aryl-14*H*-dibenzo[*a*.*j*]xanthenes (**45**) from the reactions of two equivalents of β-naphthol (**44**) and one equivalent of various aromatic aldehydes (**1**) using 10 mol% TBAB as catalyst under solvent-free conditions at 125 °C (Scheme 24). All the reactions were completed within eighty minutes and afforded 81–96% yields. Using TBAB as catalyst, the same reactions when carried out under microwave-irradiated conditions produced 78–95% yields within just six minutes. Products were isolated by simple crystallization in ethanol.

### 3.8. Synthesis of 1,8-Dioxo-Octahydroxanthenes

**Scheme 25:**
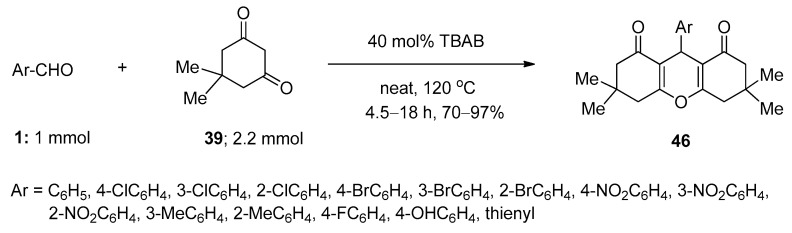
TBAB-catalyzed synthesis of 1,8-dioxo-octahydroxanthenes under neat conditions.

**Scheme 26:**
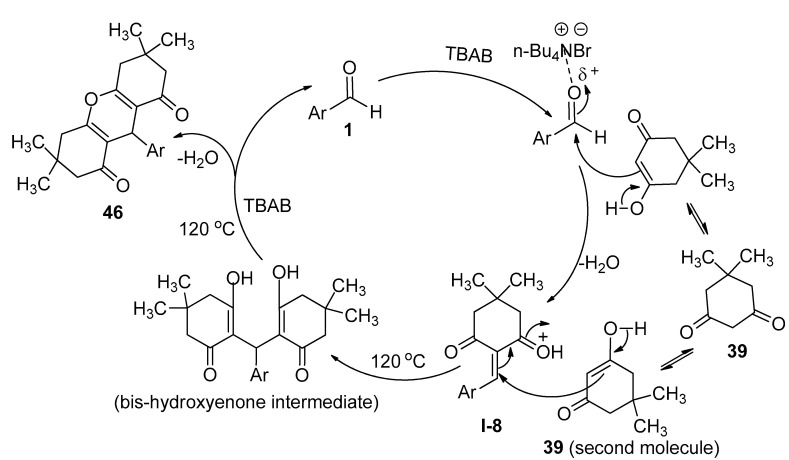
Plausible mechanism of TBAB-catalyzed synthesis of 1,8-dioxo-octahydroxanthenes under neat conditions.

Recently, 1,8-dioxo-octahydroxanthenes have gained considerable attention due to their potent pharmacological activities, such as anti-cancer, anti-bacterial, anti-microbial, and enzyme inhibitory properties [104]. Ezabadi et al. [105] synthesized a series of 1,8-dioxo-octahydroxanthene derivatives (**46**) via one-pot pseudo three-component reactions between one equivalent of aromatic aldehydes (**1**) and two equivalents of dimedone (**39**) using TBAB as catalyst under solvent-free conditions at 120 °C (Scheme 25). Various substituted aldehydes afforded the desired products with excellent yields. After being activated by TBAB, aldehydes (**1**) underwent condensation with one molecule of dimedone (**39**) and produced the corresponding intermediate **I-8** which on further attack by the second molecule of dimedone (**39**) afforded the desired product **46** at 120 °C (Scheme 26).

## 4. Applications of TBAB for the Synthesis of Bioactive *N-* as well as *O*-Heterocycles

### 4.1. Synthesis of Oxazolo[4,5-c]pyridine

**Scheme 27:**
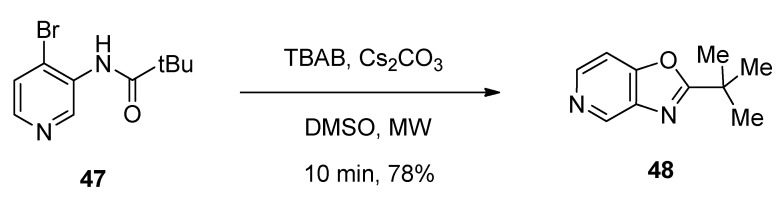
TBAB-catalyzed synthesis of oxazolo[4,5-*c*]pyridine derivatives under microwave-assisted conditions.

Tjosaas et al. [106] designed a microwave-assisted TBAB-catalyzed facile protocol for the efficient synthesis of oxazolopyridine 2-*tert*-butyl-oxazolo[4,5-*c*]pyridine (**48**) via the cyclization of 4-bromo-3-pivaloylaminopyridine (**47**) in the presence of cesium carbonate as base. The reaction was completed within just ten minutes and afforded 78% yield (Scheme 27).

### 4.2. Synthesis of Pyran-Fused Spirooxindoles

**Scheme 28:**
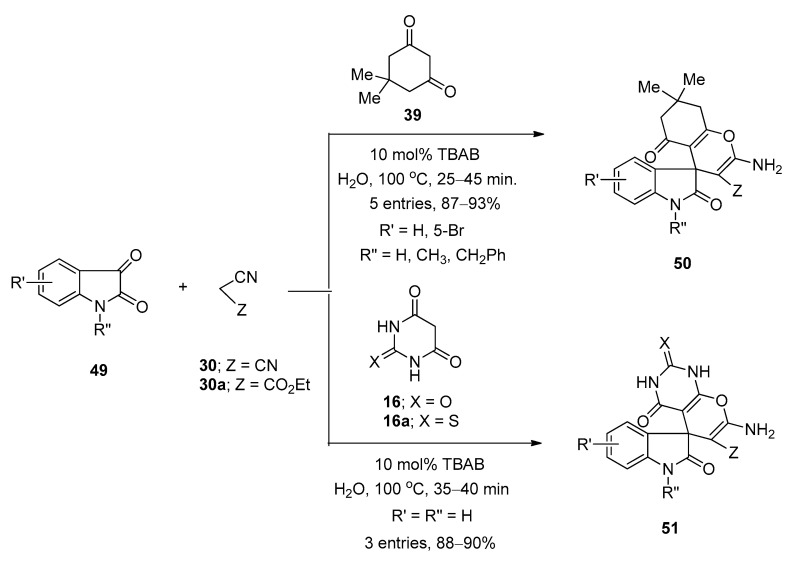
TBAB-catalyzed synthesis of spiro[(4*H*)-5,6,7,8-tetrahydrochromene-4,3′-(3′*H*)-indol]-(1′H)-2′-ones and spiro[(3′*H*)-indol-3′,4,4(*H*)-5,6,7,8-tetrahydropyrano(2,3-d)pyrimidine]-(1′*H*)-2′-ones.

**Scheme 29:**
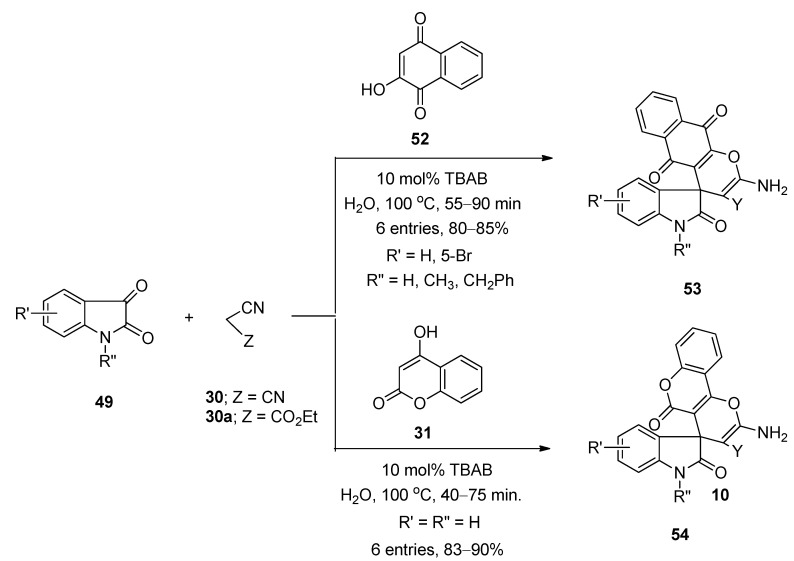
TBAB-catalyzed synthesis of spiro[(3′*H*)-indol-3′,4,4(*H*)-benzo(*g*)chromen]-(1′*H*)-2′-ones and spiro[(3′*H*)-indol-3′,4,4(*H*)-pyrano(2,3-*c*)chromen]-(1′*H*)-2′-ones.

In 2012, Mobinikhaledi et al. [107] prepared a series of structurally diverse pyran-fused spirooxindole derivatives using TBAB as catalyst in water under reflux conditions at 100 °C. They prepared spiro[(4*H*)-5,6,7,8-tetrahydrochromene-4,3′-(3′*H*)-indol]-(1′*H*)-2′-one derivatives (**50**) via one-pot three-component reactions of isatins (**49**), malononitrile (**30**) or ethyl 2-cyanoacetate (**30a**), and dimedone (**39**) (Scheme 28). Synthesis of spiro[(3′*H*)-indol-3′,4,4(*H*)-5,6,7,8-tetrahydropyrano(2,3-*d*)pyrimidine]-(1′*H*)-2′-one derivatives (**51**) was achieved from the same reactions by using barbituric acid derivatives (**16**,**16a**) instead of dimedone (**39**) (Scheme 28). Under the same optimized reaction conditions, they also prepared a number of spiro[(3′*H*)-indol-3′,4,4(*H*)-benzo(*g*)chromen]-(1′*H*)-2′-ones (**53**) and spiro[(3′*H*)-indol-3′,4,4(*H*)-pyrano(2,3-*c*)chromen]-(1′*H*)-2′-one derivatives (**54**) from the reactions of isatins (**49**), malononitrile (**30**) or ethyl 2-cyanoacetate (**30a**), and 2-hydroxynaphthalene-1,4-dione (**52**) or 4-hydroxycoumarine (**31**), respectively (Scheme 29). Almost all the reactions were completed within one hour and afforded excellent yields. Simple reaction profile, water as solvent, high atom economy, one-pot three-component synthesis, and environmentally benign nature are some of the major advantages of this developed protocol.

### 4.3. Synthesis of 1-Trifluoromethyl-3-Alkylidene-1,3-Dihydrofuro[3,4-b]quinolines

**Scheme 30:**
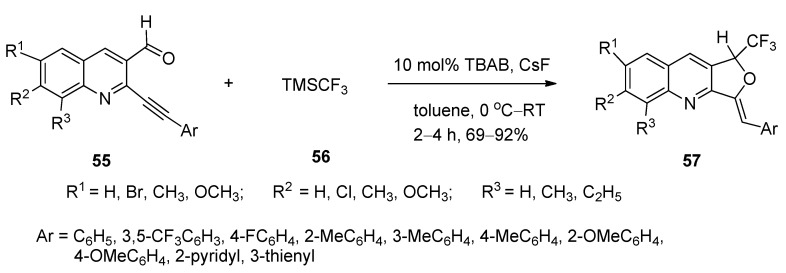
TBAB-catalyzed synthesis of 1-trifluoromethyl-3-alkylidene1,3-dihydrofuro[3,4-*b*]quinolines.

A simple, facile, and efficient method was designed for the synthesis of 1-trifluoromethyl-3-alkylidene-1,3-dihydrofuro[3,4-*b*]quinolines (**57**) via nucleophilic addition followed by cyclization reactions of *o*-arylalkynylquinoline aldehydes (**55**) with trimethyl trifluoromethyl silane (**56**) using TBAB as catalyst and cesium fluoride as base in toluene at 0 °C to ambient temperature (Scheme 30) [108]. All the products were obtained in excellent yields.

## 5. Applications of TBAB for the Synthesis of Bioactive *N-* as well as *S*-Heterocycles

### Synthesis of 1,3-Thiazine Derivatives

**Scheme 31:**
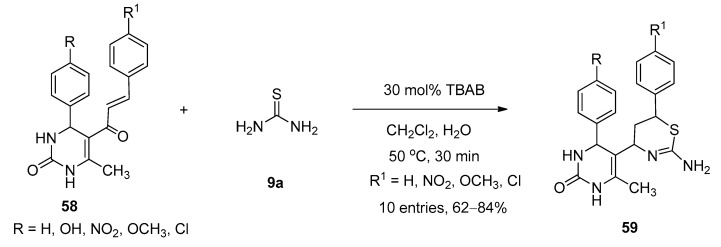
TBAB-catalyzed synthesis of 1,3-thiazine-4-yl-3,4-dihydropyrimidine-2(1*H*)-one derivatives.

**Scheme 32:**
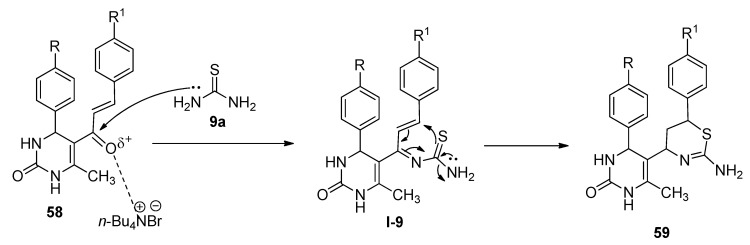
Plausible mechanism of TBAB-catalyzed synthesis of 1,3-thiazine-4-yl-3,4-dihydropyrimidine-2(1*H*)-one derivatives.

TBAB was found to be an efficient catalyst for the synthesis of thiazine derivatives. In 2018, Bankar and Dhankar [109] reported a facile and environmentally benign approach for the synthesis of 5-(2-amino-6-aryl-5,6-dihydro-4*H*-1,3-thiazine-4-yl)-3,4-dihydropyrimidine-2(1*H*)-one derivatives (**59**) from the reactions of 5-cinnamoyl-6-methyl-4-phenyl-3,4-dihydropyrimidin-2(1*H*)-ones (**58**) and excess thiourea (**9a**) in the presence of a catalytic amount of TBAB as catalyst in dichloromethane-water as biphasic solvent at 50 °C (Scheme 31). All the reactions were completed within just thirty minutes and afforded excellent yields. In the presence of TBAB, the ketonic carbon of **58** activated and formed the corresponding Schiff bases (**I-9**) with the reaction of thiourea (**9a**). The in-situ-generated **I-9** afforded the desired product by following the cyclization pathway shown in Scheme 32.

**Scheme 33:**
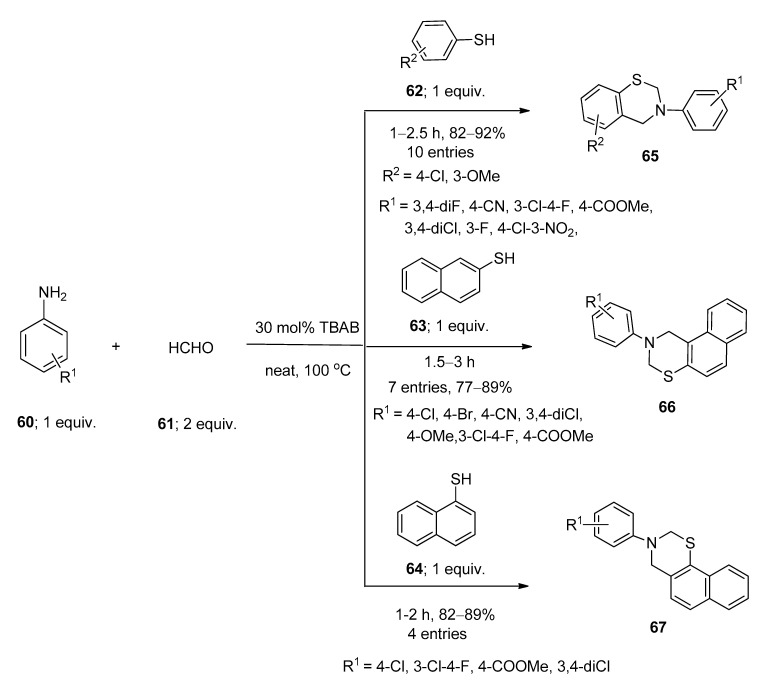
TBAB-catalyzed synthesis of benzo[*e*][1,3]thiazines, naphtho[1,2-*e*][1,3]thiazines and naphtho[2,1-*e*][1,3]thiazine derivatives.

**Scheme 34:**
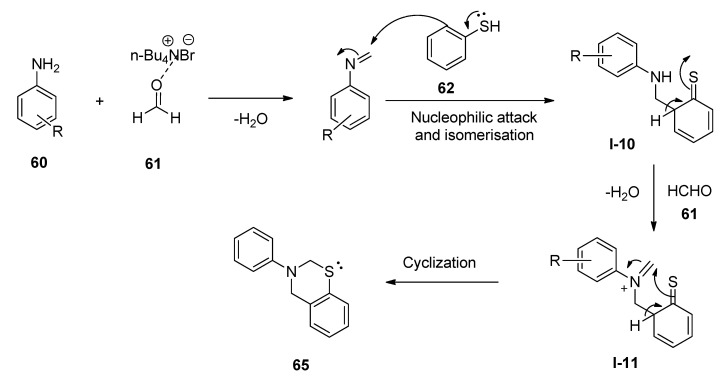
Plausible mechanism of TBAB-catalyzed synthesis of benzo[*e*][1,3]thiazines, naphtho[1,2-*e*][1,3]thiazines and naphtho[2,1-*e*][1,3]thiazine derivatives.

In the same year, Khurana and his group [110] reported another TBAB-catalyzed facile and convenient protocol for the efficient synthesis of a series of novel benzo[*e*][1,3]thiazines (**65**) via one-pot pseudo four-component reactions between one equivalent of substituted anilines (**60**), two equivalents of formaldehyde (**61**), and one equivalent of thiophenols (**62**) under solvent-free conditions at 100 °C (Scheme 33). Under the same optimized conditions, a number of naphtho[1,2-*e*][1,3]thiazine derivatives (**66**) were also synthesized from the reactions between anilines (**60**), formaldehyde (**61**), and 2-thionaphthol (**63**), whereas reactions between anilines (**60**), formaldehyde (**61**), and 1-thionaphthol (**64**) afforded the corresponding naphtho[2,1-*e*][1,3]thiazine derivatives (**67**) with excellent yields (Scheme 33). Use of a metal-free catalyst, excellent yields, broad substrate scope, and solvent-free conditions are some of the major advantages of this protocol. TBAB facilitated the formation of Schiff bases from the reactions of anilines (**60**) and formaldehyde (**61**), which on further reaction with thiophenol yielded the intermediate **I-10**. The formation of another intermediate **I-11** was accomplished via the reaction of **I-10** with the second molecule of formaldehyde. Cyclization followed by aromatization of **I-11** yielded the desired products **65** (Scheme 34).

## 6. Conclusions

Recently, TBAB has been regarded as an efficient, environmentally benign, and versatile phase-transfer catalyst. As a result, a huge number of TBAB-promoted protocols are available in the literature. In this review, we have highlighted the catalytic efficiency of this fascinating catalyst for the synthesis of various biologically promising heterocycles. It was evident the synthesis of 1,4-dihydropyridines, 2-substituted imidazolines, 2,4,5-triaryl imidazoles, 1,5-benzodiazepine derivatives, 4-phenyl-1,3-dioxolan-2-one, 5-substituted 1*H*-tetrazoles, 1,3-dihydrobenzimidazol-2-ones, 3,4-dihydropyrano[*c*]chromene, 3-nitro-2*H*-chromenes, 1,3-thiazines, and many other heterocyclic scaffolds can be achieved under mild reaction conditions by using TBAB as catalyst.
